# Natural Language Processing for Digital Health in the Era of Large Language Models

**DOI:** 10.1055/s-0044-1800750

**Published:** 2025-04-08

**Authors:** Abeed Sarker, Rui Zhang, Yanshan Wang, Yunyu Xiao, Sudeshna Das, Dalton Schutte, David Oniani, Qianqian Xie, Hua Xu

**Affiliations:** 1 Emory University, Atlanta, GA, USA; 2 University of Minnesota, Minneapolis, MN, USA; 3 University of Pittsburgh, Pittsburgh, PA, USA; 4 Cornell University, New York, NY, USA; 5 Yale University, New Haven, CT, USA

**Keywords:** Deep Learning, Large Language Models, Natural Language Processing, Medical Informatics

## Abstract

**Objectives** : Large language models (LLMs) are revolutionizing the natural language pro-cessing (NLP) landscape within healthcare, prompting the need to synthesize the latest ad-vancements and their diverse medical applications. We attempt to summarize the current state of research in this rapidly evolving space.

**Methods** : We conducted a review of the most recent studies on biomedical NLP facilitated by LLMs, sourcing literature from PubMed, the Association for Computational Linguistics Anthology, IEEE Explore, and Google Scholar (the latter particularly for preprints). Given the ongoing exponential growth in LLM-related publications, our survey was inherently selective. We attempted to abstract key findings in terms of (i) LLMs customized for medical texts, and (ii) the type of medical text being leveraged by LLMs, namely medical literature, electronic health records (EHRs), and social media. In addition to technical details, we touch upon topics such as privacy, bias, interpretability, and equitability.

**Results** : We observed that while general-purpose LLMs (e.g., GPT-4) are most popular, there is a growing trend in training or customizing open-source LLMs for specific biomedi-cal texts and tasks. Several promising open-source LLMs are currently available, and appli-cations involving EHRs and biomedical literature are more prominent relative to noisier data sources such as social media. For supervised classification and named entity recogni-tion tasks, traditional (encoder only) transformer-based models still outperform new-age LLMs, and the latter are typically suited for few-shot settings and generative tasks such as summarization. There is still a paucity of research on evaluation, bias, privacy, reproduci-bility, and equitability of LLMs.

**Conclusions** : LLMs have the potential to transform NLP tasks within the broader medical domain. While technical progress continues, biomedical application focused research must prioritize aspects not necessarily related to performance such as task-oriented evaluation, bias, and equitable use.

## 1. Introduction

Language models encode characteristics of a language in a machine-readable form—as numeric vectors. Over approximately the last decade, two major advances in language modeling have transformed how they are used in natural language processing (NLP) research and applications:

- the ability to capture word- or phrase-level semantics (meanings) in the form of dense vectors (semantically similar lexical expressions appear close to each other in a vector space) [ 1 ]; and, later;
- the ability to capture contextual variations in meanings via transformer-based models [ 2 ].

The transition from (1) to (2) was particularly impactful—pretrained transformer models enabled systems to advance the state-of-the-art in many standardized NLP tasks [ 3 , 4 ]. Broadly speaking, current state-of-the-art language models attempt to capture probability distributions over sequences of words in a given language. They do this by learning patterns and relationships between words from large amounts of text-based data. Popular mechanisms for learning such patterns include masked language modeling (e.g., predicting masked words from their contexts), and autoregressive pretraining [ 5 ] (e.g., next word prediction). Since such pretraining methods are self-supervised, and text-based data are abundant, it is possible to learn powerful representations (e.g., BERT [ 2 ] and GPT-3 [ 6 ]). Starting with BERT, the ability to further train existing pretrained transformer models led to the creation of many models—some customized for texts in restricted domains, such as the medical domain [ 7 , 8 ]. Within this period that observed unprecedented advances in language modeling, early advances were primarily in encoder-only models, and more recent advances have been in encoder-decoder or decoder-only (generative) models [ 9 ].

**Figure 1.**
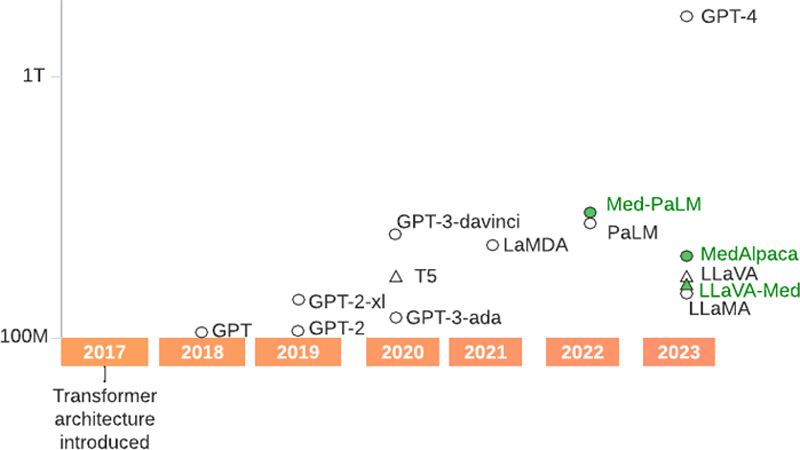
Emergence of larger large language models (LLMs) over time. The vertical axis (parameter size) is represented in the log scale. For models with multiple size variants, only the smallest and largest are shown, for visual clarity. LLMs in green depict those pretrained on biomedical datasets. Triangles are used to depict generative models with an encoder-decoder architecture. All other generative models are decoder-only.

Over the last five years, the concept of large in terms of the size of a language model has evolved rapidly ( Figure 1 ). ‘Large’ in large language models (LLMs) refers to the size of the parameters learned by these language models, which are typically measured in billions, and often trillions, these days. More parameters often allow a model to capture a broader range of linguistic patterns and relationships within the data it was trained on, which can enhance its ability to process and generate texts. Consequently, larger models also generally perform better in NLP tasks. In 2019, the largest model, to the best of our knowledge, was T5 [ 10 ] with up to 11 billion parameters. In comparison, GPT-4 is speculated to have over a trillion parameters. Among open-source alternatives, Meta's LLaMa2 has 70 billion parameters [ 11 ]. The utility of LLMs has led to their adoption in biomedical research and application, and some LLMs have been customized or tuned specifically for medical text. In the following sections, we provide an overview of the development and use of LLMs, specifically generative ones, within biomedical NLP. The rest of this chapter is organized as follows: in section 2, we outline the training of biomedical LLMs, currently available biomedical LLMs, and their uses; in sections 3 to 5, we highlight the use of LLMs for distinct types of medical texts—literature, electronic health records (EHRs), and social media, respectively; in section 6, we discuss topics such as bias, reproducibility, and privacy; and we conclude the chapter in section 7 by outlining key limitations and future directions for medical LLMs.

## 2. Adapting LLMs for the Medical Domain

### 2.1. Training Medical LLMs

Although generic LLMs like ChatGPT 1 and GPT-4 [ 12 ] have demonstrated their robustness in diverse contexts, their performance on medical texts is often suboptimal [ 13 , 14 ], perhaps due to insufficient medical domain-specific training data [ 15 , 16 ]. This gap has catalyzed the emergence of medical-specific LLMs that leverage medical big data to optimize domain-specific performance. Three strategies are typically employed for creating medical domain-specific LLMs [ 17 , 18 ]:

- Pretraining from scratch [ 19 ]: This approach requires pretraining a new medical LLM from the ground up using a vast corpus of medical data, employing the transformer architecture [ 20 ]. Tailor-made for medical applications, these models can develop a deep and nuanced understanding of medical language and contexts. However, this strategy requires extensive resources in terms of data and computational power;
- Continued pretraining [ 21 ]: This strategy involves taking a pre-existing, robust general LLM and further training it on medical-specific data. The key is to infuse the general model with enough medical data so that it can effectively represent/encode and generate medical information. Continued pretraining tasks employ the same strategies as the base models, which can be computationally intensive. Due to the importance and popularity of continued pretraining, particularly for domain adaptation, computationally efficient strategies such as low-rank adaptation (e.g., LoRA) have been proposed [ 22 ];
- Instruction tuning [ 23 ]: This method fine-tunes general LLMs for the medical domain using instruction tuning data, improving model performance for medical tasks by providing specific instructions. The training data might include task-based instructions, scenario-based queries, or decision-making processes in a medical context. The effectiveness of instruction tuning heavily depends on the quality and variety of the instructional data.

### 2.2. Emergence of Medical LLMs

Encoder-only transformer models like BERT [ 2 ] led the way to the current generative models we refer to as LLMs. Early adaptation of transformer models to the medical domain came through the release of models like BioBERT [ 7 ], PubMedBERT [ 38 ], and ClinicalBERT [ 39 ]. BioBERT was pretrained on the original BERT model using 18 billion words from PMC and PubMed, PubMedBERT was pretrained from a randomly initialized BERT model and using the entirety of PubMed data, and ClinicalBERT [ 39 ] was pretrained on a multi-center EHR dataset of diabetes patients. Explorations of the scaling effects for BERT and GPT-2-style models with increasingly large numbers of model weights, up to 8.3 billion, demonstrated that performance increased for both model types at extremely large sizes [ 40 ]. This work was followed by pretraining using PubMed data from newly initialized models and models pretrained on general corpora [ 41 ]. The resulting BioMegatron 345M parameter model showed stronger performance than PubMedBERT on multiple standardized NLP tasks. These works paved the way for new-age generative LLMs with billions of parameters.

We summarize representative LLMs in Table 1 . Among them, MedPaLM [ 24 ], MedPaLM2 [ 42 ], ChatDoctor [ 25 ], MedAlpaca [ 26 ], Clinical Camel [ 28 ], AlpaCare [ 34 ], LLaVA-Med [ 31 ], MMedLM 2 [ 37 ] and Med-Flamingo [ 33 ] adopted the instruction tuning strategy. MedPaLM and MedPaLM2, which utilize the PaLM architecture with 540 billion parameters, and PaLM2 as the backbone, have been shown to be particularly effective in medical question-answering (QA) tasks. AMIE [ 34 ] also uses PaLM2 as the backbone model, and is specifically fine-tuned for clinical applications, based on medical QA and clinical texts. However, its large parameter size and closed-source nature make it challenging for widespread deployment, particularly in resource-constrained settings. Models such as ChatDoctor and MedAlpaca, based on the open-sourced LLM LLaMA [ 11 , 43 ], offer a more practical balance. With a focus on QA and conversations, they provide versatility while being less computationally intensive, making them more accessible for diverse medical applications.

**Table 1.** A detailed comparison of medical LLMs available at the time of writing. This comparative analysis spans multiple dimensions, providing a holistic view of each model's characteristics and capabilities. Key aspects of comparison include: backbone model, model size, training strategy, domain-specific data used and data size, accessibility, language, and release date. IFT: instruction fine-tuning; CPT: continued pretraining; SPT: pretraining from scratch; RLMF: reinforcement learning with mixed feedback; QA: question answering. For MedPaLM2, there is no information about the data size and model size of its backbone model PaLM2 (the same backbone model for AMIE). Models without explicit license terms that can be accessed are denoted by a checkmark while models without access are denoted by ‘X’. Consumer-Mediated Health Information Exchange: Authorize Direct Transmission.

Model	Backbone	Size	Strategy	Data	Data Size	Modality	License Access	Language	Release date
MedPaLM [ 24 ]	PaLM	540B	IFT	QA	-	Text	X	English	12/26/22
ChatDoctor [ 25 ]	LLaMA	7B	IFT	Conversation	100K	Text	Apache 2.0	English	03/24/23
MedAlpaca [ 26 ]	LLaMA	7B, 13B	IFT	QA	160K	Text	GNU GPL v3.0	English	04/14/23
PMC-LLaMA [ 15 ]	LLaMA, LLaMA2	7B, 13B	CPT+IFT	Literature, book, conversation, QA, knowledge graph	79B, 514K	Text	Apache 2.0	English	04/25/23
MedPaLM2 [ 27 ]	PaLM2	-	IFT	QA	-	Text	X	English	05/16/23
Clinical Camel [ 28 ]	LLaMA2	13B, 70B	IFT	Conversation, QA	104K	Text	GNU Affero GPL v3.0	English	05/19/23
GatorTronGPT [ 29 ]	GPT-3 architecture	5B, 20B	SPT	General and clinical text	82B	Text	Apache 2.0	English	05/24/23
HuatuoGPT [ 30 ]	Baichuan, Ziya	7B, 13B	IFT+RLMF	Conversation, QA	-	Text	Apache 2.0	Chinese	05/25/23
LLaVA-Med [ 31 ]	LLaVA	13B	IFT	biomedical image-text	630K	Text, image	Microsoft ResearchLicense	English	06/01/23
Clinical-LLaMA [ 32 ]	LLaMA2	7B	CPT	MIMIC-IV	-	Text	X	English	07/12/23
Med-Flamingo [ 33 ]	Flamingo	9B	IFT	General and biomedical image-text	>0.01B	Text, image	√	English	07/27/23
AlpaCare [ 34 ]	LLaMA2	7B, 13B	IFT	Biomedical conversation	52K	Text	Apache 2.0	English	10/23/23
Meditron [ 16 ]	LLaMA2	7B, 70B	CPT	General and biomedical text	48B	Text	Apache 2.0	English	11/27/23
AMIE [ 35 ]	PaLM2	-	IFT	QA, clinical text	110K	Text	X	English	01/11/24
Me LLaMA [ 36 ]	LLaMA2	13B, 70B	CPT+IFT	QA, clinical text	129B, 214K	Text	PhysioNet Credentialed HealthData License 1.5.0	English	02/20/24
MMedLM2 [ 37 ]	InternLM, BLOOM	7B	IFT	QA, clinical text	25.5B	Text	cc-by-4.0	Multilingual	02/26/24

GatorTron [ 44 ] and GatorTronGPT [ 29 ] represent the few medical LLMs that have been trained from scratch. GatorTronGPT is based on the GPT-3 [ 45 ] framework, and it was trained using 200 million clinical notes, and 124 NVIDIA DGX nodes.

PMC-LLaMA [ 15 ], Clinical-LLaMA [ 28 ], Meditron [ 16 ] and Me LLaMA [ 36 ] represent models using the continued pretraining, or domain-adaptive pretraining approach. These models utilize foundation LLMs that are further enhanced with specific medical knowledge. All these models use LLaMA2 as their backbone, given its strong performance in general domain tasks and open-source nature. PMC-LLaMA and Me LLaMA further combine this approach with instruction tuning, demonstrating an effective, hybrid strategy to adapt the LLaMA2 model to the medical domain.

### 2.3. Characteristics of Medical LLMs

In the dynamic landscape of medical LLMs, a nuanced understanding of their varied characteristics is crucial for effective application. The distinctions between open and closed-source models, parameter size, and training strategies offer insights into their adaptability and utility in different medical settings.

Open vs. Closed Source. The contrast between open-source models such as ChatDoctor and MedAlpaca and closed-source models like MedPaLM presents a fundamental choice in the medical community. Open-source models foster collaboration, enabling extensive enhancement and personalization. Conversely, closed-source models may offer better professional support, user interfaces, and performance, but confine their development within proprietary boundaries, thus restricting their availability and flexibility.

Parameter Size and Computational Complexity. Parameter size is a pivotal factor in the selection of medical LLMs. Large models like MedPaLM (540 billion parameters) offer in-depth understanding and complex reasoning capabilities. However, their deployment demands substantial resources, making them less feasible for constrained environments. Conversely, smaller models in the 7B-13B range, such as MedAlpaca and AlpaCare, strike a balance between efficiency and effectiveness, facilitating easier deployment.

Domains, Modality, Language and Usage. In the realm of medical LLMs, the majority of studies focus on published biomedical literature text, often overlooking clinical data and tasks. For example, models like PMC-LLaMA and AlpaCare excel in the biomedical literature domain with robust instruction-following capabilities. Meditron, though high-performing, lacks the instruction-following ability, limiting its scope in instruction-driven tasks. Relatively fewer models, such as Clinical-LLaMA and GatorTronGPT, focus on the clinical domain. GatorTronGPT, trained on 82 billion clinical texts and available in 5B and 20B parameter model sizes, demonstrates potential, yet its generalization abilities are constrained by its size. Meanwhile, Clinical-LLaMA, primarily trained on MIMIC-IV's limited clinical texts, concentrates on classification tasks, not fully exploiting the capabilities of LLMs in a variety of clinical settings. Most medical LLMs focus on textual data, but emerging models like Med-Flamingo introduce multimodal capabilities (e.g., via fine-tuning on image caption data), incorporating image analysis which is invaluable in fields like radiology or pathology. The language focus is predominantly English, with exceptions like HuatuoGPT, which is tailored for Chinese, and MMedLM 2, which supports six major languages: Japanese, Spanish, French, Russian, English, and Chinese, indicating a growing trend towards accommodating linguistic diversity in medical LLMs. The choice between these models should be based on the specific needs of biomedical versus clinical applications, data modality, and the complexity of tasks involved.

### 2.4. Evaluation of LLMs on Biomedical Tasks

The Biomedical Language Understanding Evaluation (BLUE) framework was proposed to facilitate a biomedical counterpart to the popular General Language Understanding Evaluation (GLUE) framework [ 46 ]. Comprising five tasks on ten biomedical corpora, resources from BLUE have been partially adopted by some models as part of their evaluation [ 47 , 48 ]. Since the tasks on which medical LLMs are trained are diverse, the evaluation metrics vary. Due to this, there is currently no objective measure to help select an LLM for a specific medical task beyond choosing models that have been specifically pre-trained on domain-specific text. Human evaluation of model outputs has been used in addition to general language model evaluation metrics like BLEU and ROUGE [ 49 , 50 ] for medical text summarization. Similarly, concept extraction from EHRs has been studied through qualitative evaluation [ 51 ]. Akin to the BLUE framework, the MultiMedQA framework comprising nine tasks for quantitative evaluation in addition to additional tasks for qualitative evaluation has been proposed [ 52 ]. The MedEval testbed focuses on covering a wider variety of human body parts, as compared to existing frameworks that prioritize task coverage [ 53 ].

Beyond the English language, MedBench [ 54 ] and MMedBench [ 37 ] have been introduced as evaluation frameworks for Chinese and multilingual medical LLMs, respectively. BioMistral 2 , a collection of open-source pretrained biomedical LLMs, also features a multilingual evaluation framework in seven languages, although the drawbacks of auto-translation remain as they translate an English benchmark into seven languages. The framework CRAFT-MD 3 focuses exclusively on the task of conversational reasoning for evaluating medical LLMs. Since evaluation of LLMs is an evolving area of research, just like LLMs themselves, novel evaluation strategies (such as hallucination evaluation [ 55 ]) are actively being proposed, and we anticipate a trend of more standardized and converging benchmarking of medical LLMs in the future.

## 3. LLMs for Medical Literature

Most training and application of medical LLMs have focused on text from the medical literature. In this section, we briefly review key NLP tasks, highlight some of the most important models that were trained on the biomedical literature, discuss their applications to specific tasks, and address challenges associated with their use.

### 3.1. LLM Applications in Biomedical Literature

Several NLP tasks have relevance when applying LLMs to the biomedical literature. Named Entity Recognition (NER) is the identification of words that belong to a class of interest (drugs, symptoms, etc.). Relation extraction is identifying relationships between named entities from the literature (e.g., drug A treats condition Z). Literature-based discovery is the prediction of new relationships between entities using existing entities and relationships extracted from a corpus. Other tasks include question answering (QA) where the model responds to a query given some biomedical text as input, information retrieval to identify and extract relevant information from text, and summarization to provide a concise description of larger biomedical text.

#### 3.1.1. Information Extraction

LLMs have shown moderate to good, though not state-of-the-art, performance on NER. For example, a GPT model augmented with a biomedical knowledge graph was able to obtain respectable F1 scores [ 56 ]. The same study also showed that LLMs were able to achieve impressive scores for zero-shot NER. There is some potential for LLMs to be used for drug repurposing, a literature-based discovery task, as recent research has demonstrated that even the generic GPT-4 model can perform well on this task [ 57 ] by fine-tuning the model with prompts that are augmented with knowledge from a biomedical knowledge graph. Recently, there have been explorations into using LLMs, such as MedLLaMA, for relation extraction and knowledge graph construction [ 58 ]. While LLMs can be used for information extraction, a recent survey found that PubMedBERT often outperformed GPT-3.5 and GPT-4 on information retrieval-focused tasks [ 59 ]. These studies demonstrated that there is room for improvement on these tasks for LLMs.

#### 3.1.2. Question Answering

LLMs have demonstrated exceptional performance on a wide variety of QA-focused tasks. ChatDoctor, PMC-LLaMA, MedAlpaca [ 26 ] have all demonstrated strong performance on a range of biomedical QA datasets. This suggests that these models, tuned on the literature, may be useful for information retrieval and for reducing the time physicians need to spend looking through PubMed to find answers to their questions. MedAlpaca, in particular, has achieved state-of-the-art zero-shot performance in answering questions from the three parts of the United States Medical Licensing Exam series [ 26 ].

#### 3.1.3. Information Retrieval

Combining LLMs with external databases for information retrieval and response generation, retrieval augmented generation, has shown promise in improving the specificity of answers provided to users [ 60 ]. As discussed later in this survey paper, hallucinations are a problem with any LLM but can be particularly hazardous in a medical context where a hallucination could lead to erroneous advice generation. However, this can be mitigated by the use of knowledge graphs to enhance reasoning and ground LLMs to decrease the likelihood of hallucinations [ 57 ].

#### 3.1.4. Summarization

Recent work has shown that LLMs can effectively be fine-tuned on small amounts of domain-specific data to produce high-quality summarizations of biomedical research abstracts [ 61 ]. A sample of 175 abstracts from the specialized COVID-19 dataset, CORD-19 [ 62 ], was used with GPT-3.5 and Davinci (the initial model of the GPT-3 series) to produce a dataset of synthetic prompts that were used to successfully fine-tune other LLMs to produce summarizations of abstracts.

## 4. LLMs for Electronic Health Records

Large-scale data mining from free text notes in EHRs and extracting meaningful information has been a challenge for NLP research over the years, and LLMs present substantial opportunities to move the state-of-the-art forward. Consequently, LLMs have found widespread use in the context of EHRs. Here, we outline a subset of key applications of LLMs for EHR data.

### 4.1. Clinical Decision Support Systems

Clinical Decision Support Systems (CDSSs) enhance medical decision-making by providing targeted clinical knowledge, patient information, and other health information [ 63 , 64 ]. Recently, LLMs have shown great potential as core CDSS components. LLMs trained or fine-tuned on texts from EHRs can capture high-quality representations of input texts that can be used for tasks such as diagnosis prediction [ 65 ], clinical trial outcome measurement [ 66 ], and in-hospital mortality prediction [ 67 ], to name a few. In-context learning capabilities [ 6 , 68 ] make generative LLMs inherently interactive, allowing for direct conversations and QA sessions between physicians and LLMs. Such interactive artificial intelligence (AI) systems built on NLP technology was largely infeasible prior to the recent advances in generative LLMs. Chatbots have become a common component in new-age CDSS designs with generative LLM integration [ 69 , 70 ]. A number of studies have already demonstrated the effectiveness of using such chatbots for clinical decision support, particularly illustrating their potential to improve productivity in healthcare settings and facilitate better health outcomes [ 29 , 71 , 72 ]. While research in this space is still early, the overwhelmingly promising results suggest that chatbots based on generative LLMs and trained on EHR texts will see significant adoption across healthcare systems. In terms of specific applications, text-to-text LLMs have found use in chest X-ray report summarization [ 73 ], summarization and classification of medical dialogues [ 74 ], and diagnostic reasoning [ 75 ], to name a few. Due to the many possible applications of LLMs on EHR texts, as mentioned earlier in this article, a number of models have been proposed that have been trained or fine-tuned on EHR-specific data—the most notable of them perhaps being GatorTron [ 44 ].

### 4.2. Revenue Cycle Optimization

Revenue Cycle Management (RCM) is a critical process for healthcare organizations that typically consists of pre-encounter, intra-encounter, and post-encounter steps [ 76 ]. Revenue Cycle Optimization (RCO) analyzes each of these steps to identify areas that can increase revenue, reduce expenses, and improve cash flow. Therefore, RCO helps streamline RCM, resulting in efficient billing processes, reduced claim denials, and timely reimbursements, which can significantly improve the overall quality of care, ultimately driving better health outcomes [ 77 ]. Traditionally, RCO is a manual, highly complex endeavor with plenty of opportunities to augment human decision-making with AI.

Generative LLM-based chatbots can help with pre-encounter and inter-encounter tasks, including appointment scheduling, patient registration, and prior authorization. Given the politeness and empathy that can be infused into these chatbots in simulated healthcare settings [ 78 ], their use in patient interactions holds great promise. According to a 2019 report [ 79 ], payment errors amount to hundreds of billions of dollars in unnecessary spending annually. To this end, NYUTron has shown that EHR-trained generative LLMs can predict insurance denials, reducing billing complexity and payment errors [ 80 ].

### 4.3. Translational Research

Translational research is defined as “the process of applying ideas, insights and discoveries generated through basic scientific inquiry to the treatment or prevention of human disease” [ 81 ]. The application of AI-driven methods to translational research presents a clear opportunity for multidisciplinary collaborations, with teams comprising diverse domain experts, such as computer scientists, mathematicians, and clinicians. Indeed, many successful translational research efforts have had authors with wide-ranging research backgrounds [ 82 83 84 ]. This trend is likely to continue in the case of LLMs trained on EHRs, with researchers discovering novel applications to medicine [ 85 ]. Clinical trials, the gold standard for evaluating new treatments, are critical in translational research. Feasibility studies before conducting clinical trials leverage LLMs to analyze patient records, aiding in the assessment of a trial's practicality by quickly determining if sufficient suitable participants are available. This accelerates the early stages of research. LLMs can also analyze EHRs to extract and normalize medical concepts to identify potential trial participants. Systems like EliIE [ 86 ] could be enhanced by LLMs, facilitating patient cohort definition and even enabling in silico trials. Moreover, LLMs can play a crucial role in patient recruitment, swiftly analyzing clinical documents in EHRs to match patients with appropriate trials, thereby streamlining recruitment and ensuring that trial opportunities are maximized. Lastly, LLMs can aid in analyzing clinical trial criteria and matching patients with the most suitable trials, offering patients access to the latest treatment options, for example, TrialGPT [ 87 ].

## 5. LLMs for Health-related Social Media Data

Social media chatter contains an abundance of health-related information as subscribers often discuss such topics with their peers. A small survey, for example, showed that over 80% of cancer patients conducted disease-related communication with others over social media [ 88 ]. Often, health-related information available from social media are not available from any other sources [ 89 ]. Knowledge relevant to health can be acquired from social media in close to real-time [ 90 ], at scale, and often from hard-to-reach populations [ 91 ]. NLP of health-related social media texts has proven difficult (relative to, for example, text from medical literature) over the years for various data-level characteristics, such as misspellings and colloquial and evolving language [ 92 ]. Despite the difficulties associated with NLP of social media data, recent progress has demonstrated their utility for complex tasks, such as targeted public health surveillance [ 93 ] and behavior analysis [ 94 ].

Literature on the application of generative LLMs for social media based health-related tasks is sparse, with only a handful of papers published to date. This is unsurprising since applications of new NLP technology within the health space typically target domain-specific text from sources other than social media such as medical literature. Once these methods are mature, they are applied and often customized for social media texts, which represent an array of additional NLP challenges. We outline some important recent research contributions, noting that most language models in application today are still transformer-based encoder models, which outperform new-age generative models in benchmarking experiments [ 95 ].

### 5.1. LLMs for Mental Health

Social media sources encapsulate a trove of knowledge regarding mental health, and, consequently, a substantial chunk of health-related NLP research involving social media data has focused on mental health-related topics [ 96 ]. With the emergence of new-age LLMs, the trend has been no different. A very recent contribution is the MentaLLaMA model [ 97 ]. The paper introducing the model also presents the interpretable mental health instruction dataset built from social media data. The MentalLLaMA model is shown to perform close to supervised state-of-the-art discriminative models while providing reasonable explanations. Despite less than optimal performance, MentaLLaMA represents an exciting branching of open-source LLMs—the first to specifically address health-related social media text. A similar theme of using social media data for providing conversational text assistance pertaining to mental health issues is demonstrated by Psy-LLM [ 98 ]. They use data from the Chinese social media platforms Tianya, Yixinli, and Zhihu. Inevitably, many more models customized for targeted health-related topics over social media data will follow suit.

Xu et al. [ 99 ] present a comparative evaluation of zero-shot, few-shot (examples inserted into the prompt), and instruction fine-tuned LLMs on mental health datasets obtained from the social media platform Reddit. Their fine-tuned models Mental-Alpaca, based on Alpaca [ 100 ], and Mental-FLANT5, based on the T5 framework [ 10 ], outperform GPT-3.5 and GPT-4. These results demonstrate the promise of subdomain-specific LLMs in digital health.

### 5.2. LLMs for Health Surveillance

Identifying and monitoring disease outbreaks in real-time with social media has been useful for health institutions, including the World Health Organization [ 101 ]. Although the use of LLMs in infoveillance is still nascent, the ability of LLMs to extract symptoms from social media posts has already been explored [ 102 ] in the context of COVID-19. The study showed that with prompt engineering, GPT-3.5-Turbo and GPT-4 can achieve high precision and recall. The authors also reported using ChatGPT as an aid for prompt engineering to arrive at the final prompt used to compare different LLMs, including Google Bard. Such use of LLMs to aid in using other LLMs is likely to rise in the future.

### 5.3. LLMs for Combating Health Misinformation

The rapid spread of misinformation through social media is an active topic of concern among the broader healthcare community [ 101 ]. With LLMs generating text that is indistinguishable from those written by humans, there is notable risk of misuse of LLMs to generate misinformation at scale. Chen et al. [ 103 ] point out the dichotomy of LLM usage in both promoting as well as combating health-related misinformation on social media platforms. They highlight the use cases of detecting health-related misinformation by augmenting LLMs with external knowledge bases. With social media platforms integrating LLMs more closely into their ecosystem, misinformation intervention techniques such as LLM-generated factual counter-misinformation responses are on the horizon. Xiao et al. [ 104 ] take a step in this direction by creating Jennifer, a chatbot to provide expert-sourced answers to commonly asked questions about COVID-19. They deployed their chatbot on Facebook, in addition to other websites, and found that Jennifer was able to help users find more accurate answers to COVID-19-related queries. They proposed using LLMs to complement their expert-sourcing framework for faster deployment in future versions of their chatbot.

### 5.4. LLMs for Health Opinion Mining

The massive amount of opinion generated on social media platforms makes them lucrative for gauging public sentiments toward health policies as well as early detection of rapidly changing health threats like COVID-19. Tran et al. [ 105 ] utilized LLMs for COVID-19-related public opinion mining using Twitter (X) data from Japan. Although the LLMs exhibited high performance variability in zero-shot settings with different prompt designs, the study showed a useful application of LLMs in digital health.

## 6. LLMs: Challenges, Limitations, and Risks

### 6.1. Privacy and Data Security

Closed-source LLMs such as ChatGPT have seen substantial rise in popularity owing to their ease of use. These LLMs invoke API 4 calls to the model hosted off-site with possible personally identifiable information (PII) being transmitted. Some healthcare institutions have established internal guidelines to prevent such off-site transmission of data, while some have reached agreements with service providers to securely transfer PII. Much like LLMs themselves, privacy and data security standards associated with LLM use in healthcare settings are evolving. Mesko and Topol [ 106 ] highlight the need for informed patient consent for the use of LLMs in their personal healthcare management.

### 6.2. Bias and Fairness

Several studies have found that LLMs do not generalize well across distinct demographics. GPT-4 has been shown to exhibit bias when diagnosing people of different genders, races, and ethnicities [ 107 ]. Regulation of LLMs, particularly in healthcare, has been suggested [ 106 ] as a means to protect vulnerable populations from harm. The sparsity of multilingual medical LLMs is another factor contributing to geographical bias in the adoption of medical LLMs. The MMedC medical corpus covering five languages, CHIMED-GPT for Chinese, and the bilingual mixture-of-experts LLM BiMedX for Arabic medical tasks are noteworthy attempts to extend the application of LLMs in more languages. Perhaps the most impactful stride toward alleviating geographical disparities is demonstrated by Apollo [ 108 ]. Covering a population of 6 billion through six languages, Apollo is further distinguished by being lightweight.

### 6.3. Interpretability, Explainability, Transparency, and Equitable AI

A major issue hindering the adoption of LLM-based technologies in healthcare is the lack of transparency. LLMs may exhibit opacity across different dimensions: closed-source models, lack of information on training data and procedures, and limited interpretability and explainability. This lack of transparency adds to the issue of accountability, which is paramount in medicine. Although interventions such as the foundation model transparency index [ 109 ] have been proposed, the success of such approaches largely relies on their adoption.

The compute-intensive nature of large models poses challenges to equitable use of AI. For large models, it is necessary to utilize clusters of GPUs for time-efficient fine-tuning and inference. For example, at full precision, LLaMa-v2 70B requires at least 8 A100 NVDIA GPUs for tuning. Not all researchers and developers have access to the resources necessary to tune or deploy these models, which inevitably leads to inequity in research and application of LLMs in medicine. Some techniques in “TinyML” have been proposed to reduce the footprint of these large models and include techniques such as training subsets of weights (LoRA), quantization, and precision reduction [ 110 ].

### 6.4. Evaluation of LLM Performance, Reproducibilty, and Deployability

In terms of NLP tasks, multiple studies have shown that for tasks such as supervised classification and information extraction, transformer-based models still outperform LLMs when large labeled training data is available. LLMs, however, typically perform better for these tasks in low-/zero-shot settings [ 56 , 111 ]. For generative tasks such as summarization and QA, LLMs have demonstrated the greatest improvements compared to earlier methods [ 75 , 112 ]. Xu et al. [ 99 ] stress, in the context of mental health, that despite the impressive performance of LLMs, their deployability for digital health applications remains in question. For deployment in healthcare, LLMs must be evaluated on aspects beyond accuracy or other intrinsic evaluation metrics. Targeted pre- and post-deployment evaluations of fairness, bias, and interpretability are essential, along with robust strategies for effective identification and mitigation of ethical concerns [ 107 ].

Reproducibility can often be challenging to ensure since LLM outputs may be non-deterministic. This may particularly happen with API-based LLMs since the closed-source back-end models may change over time, rendering past outputs irreproducible. Additionally, open-source models may lose their initial capabilities when further tuned on new data, a problem known as catastrophic forgetting [ 21 , 113 , 114 ].

Finally, LLMs used for generative tasks pose a risk for hallucinations that can be at best annoying or at worst dangerous in the case of biomedical text generation for QA or summarization. Hallucinations can be particularly hard to predict and may remain undiscovered if models are not evaluated rigorously. The ease of use of API-based models like GPT-4 particularly make non-expert researchers (i.e., those without substantial experience in NLP research) susceptible to interpreting coherent responses generated by LLMs to be accurate information. Extensive intrinsic and extrinsic evaluations, led by researchers with substantial NLP expertise, are necessary to mitigate the dangers of hallucination.

## 7. Concluding Remarks

Notwithstanding the challenges associated with the use of LLMs in biomedical NLP tasks, there remains vast potential for integrating LLMs in research, education, and clinical care [ 115 ]. Retrieval augmented generation has been gaining popularity as a method to reduce hallucinations [ 116 ].

Prompt modification, integration of external data sources, and leveraging knowledge graphs for reasoning augmentation have also been found to mitigate hallucinations [ 117 ]. Evaluating general purpose LLMs for trustworthiness across several dimensions such as reliability, safety, and resistance to misuse has been proposed [ 118 ]. Designing trustworthiness criteria specific to healthcare systems can benefit the advancement of medical LLMs. As more effective approaches for mitigating the limitations associated with LLMs are developed, their adoption in biomedical NLP tasks is expected to increase further. We expect to see a trend of more equitable dissemination of LLM infrastructure with smaller, less resource-intensive models in the future. Biomedical subdomain-specific models, similar to MentalLlaMA for the subdomain of mental health, are also envisaged.

LLMs have brought about a new paradigm in biomedical NLP research and application, enabling us to make substantial progress on problems that appeared unsolvable even in the recent past (e.g., QA). Thus, the proliferation of LLM use in digital health is largely desirable, and the future is promising. However, it is critical to establish and implement necessary guardrails for responsible usage to mitigate the possibility of unintended harm.

## Footnotes

1 https://openai.com/blog/chatgpt [accessed: 29 Jan, 2024]

2
https://arxiv.org/abs/2402.10373

3
https://www.medrxiv.org/content/10.1101/2

4 Application Programming Interface; for communication between a server and the client/user.
